# Value-Concordant Decision Making in Patients on Prolonged Mechanical Ventilation: A Family-Centered Care Approach Using the Calgary Family Assessment and Intervention Models

**DOI:** 10.7759/cureus.99004

**Published:** 2025-12-11

**Authors:** Mutsumi Tsukamoto, Syunsuke Hayashi, Mitsunobu Toyosaki, Toshiharu Nakama

**Affiliations:** 1 High Care Unit, Yuuai Medical Center, Tomigusuku, JPN; 2 Intensive Care Unit, Yuuai Medical Center, Tomigusuku, JPN; 3 Adult Nursing, Faculty of Nursing, Daiichi University of Pharmacy, Fukuoka, JPN

**Keywords:** decision making, discharge planning, family-centered care calgary family assessment and intervention model, mechanical ventilation, spinal cord injury, value-concordant

## Abstract

Family-centered care (FCC) emphasizes collaboration with family members to build shared understanding and support decision-making. The Calgary Family Assessment Model (CFAM) and the Calgary Family Intervention Model (CFIM) provide a practical structure for translating this approach into family assessment, communication, and tailored family support. A man in his 60s with high cervical (C3/4) spinal cord injury required prolonged mechanical ventilation. Nurses integrated FCC with the CFAM and CFIM through structured family conferences using teach back and circular questioning, CFAM-guided assessment, CFIM interventions across cognitive, affective, and behavioral domains, and an option grid comparing home and facility care. This bedside implementation clarified the core value-time to talk freely with family-and reframed a binary “wean-then-home” assumption into parallel planning. The patient and family chose to discontinue weaning and transfer to a facility with flexible visitation, as this was the best goal, being value-concordant and safe. This integrated approach supported a nonhierarchical partnership and fostered trust, shared understanding, and effective decision making.

## Introduction

Family-centered care (FCC) in intensive care units (ICUs) was consolidated in the 2017 Society of Critical Care Medicine guideline across neonatal, pediatric, and adult settings, emphasizing flexible family presence and structured engagement with families [1]. Contemporary adult-ICU guidance has since updated these practices post-pandemic era, standardizing communication support and liberalizing family presence as the default when feasible [2]. As frameworks that translate partnership principles into bedside processes, the Calgary Family Assessment Model (CFAM) structures family assessment across structure, development, and function, whereas the Calgary Family Intervention Model (CFIM) aligns interventions with the cognitive, affective, and behavioral domains and emphasizes ongoing reassessment [3,4]. Within this framework, circular (reflexive) questioning shifts attention from linear causality to relational (circular) interaction patterns among family members, thereby revealing the patients’ core values and facilitating meaningful changes [5]. In time-limited ICU encounters, a standardized family interview (≤15 minutes) provides operational guidance for engaging, assessing, and intervening efficiently at the family-clinician interface [6]. Point-of-care decision aids, particularly Option Grids, make feasible alternatives explicit and support shared decision-making by structuring comparisons around patients’ frequently asked questions [7]. In the present case report, nurses coordinated an interdisciplinary team in partnership with the family as equal collaborators, within the CFAM/CFIM framework, to operationalize FCC’s participation and collaboration pillars and align choices with the patient’s articulated values.

Throughout the patient’s course from admission to the ICU through transfer to a general ward via the high care unit (HCU), FCC was maintained as the guiding approach. Structured family interviews and multidisciplinary conferences involving family participation were conducted using the CFAM/CFIM. The present case report aimed to demonstrate, in an operational and replicable manner, how a patient-centered goal aligned with an individual’s core values was identified and implemented for a patient with high cervical spinal cord injury requiring prolonged mechanical ventilation (MV). Nurses led the design of decision-support processes and integration of information across disciplines, translating FCC and CFAM/CFIM from concepts into bedside practice.

## Case presentation

The patient, a man in his 60s, sustained a C3/4 cervical spinal cord injury following a workplace fall, resulting in immediate tetraplegia. Sensory and motor functions were preserved above the level of the neck, including the face. His consciousness remained clear throughout.

On the first day after injury (DAI 1), MV was initiated in the ICU. An early tracheostomy was performed on DAI 4, and a weaning trial from MV was initiated on DAI 5. Short periods of liberation were intermittently achievable owing to preserved spontaneous respiration. However, subsequent hypoalbuminemia-related pleural effusion and alveolar hypoventilation leading to carbon dioxide narcosis precipitated repeated returns to MV. Orthopedic surgeons managed the primary spinal pathology, intensivists directed systemic management, and the RST handled ventilatory care. During the early phase of his stay in the ICU and HCU, the patient developed aspiration pneumonia and a urinary tract infection, which both resolved promptly with standard treatment. On DAI 10, body image alterations triggered psychological distress and insomnia, which led to fluctuating intake and RH, thereby reducing activity tolerance and hindering the development of weaning endurance. A multidisciplinary mental health liaison team was consulted on DAI 15.

From the earliest stage of hospitalization, the patient consistently expressed a desire to “live normally at home.” His spouse also wanted to honor this wish but felt that providing home care at his current level of dependence was not feasible, as age-related physical limitations had reduced her ability to provide hands-on care. He lived with his spouse, who was of similar age, in a two-person household before hospital admission. Their three adult children lived separately with their respective families, approximately an hour's drive away. Although they were supportive, the children were unable to provide routine caregiving should their father require long-term support because of family commitments. During hospital admission, his wife remained supportive, visiting daily and participating in his care. However, her age-related physical limitations had reduced her ability to provide hands-on care, a constraint she openly acknowledged. Discharge planning initially assumed successful liberation from MV as a prerequisite for home discharge, but repeated setbacks delayed progress.

**Figure 1:**
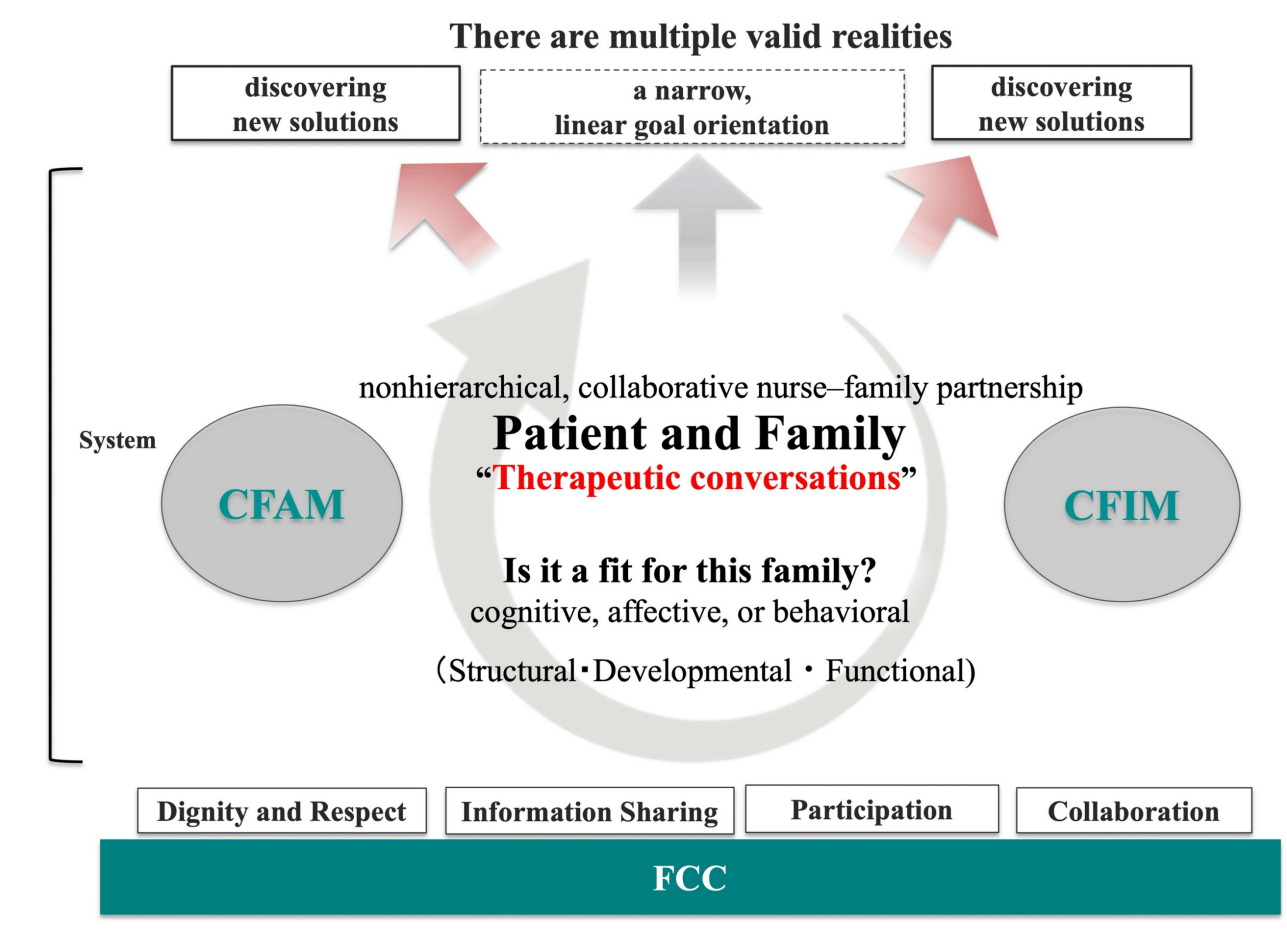
A clinical implementation model integrating family-centered care and the CFAM/CFIM A clinical implementation model based on the four concepts of FCC that restructures "therapeutic conversations" into multiple optimal goals that integrate fixed goals with patient values ​​by repeatedly conducting family assessments using CFAM and interventions using CFIM. This figure was created by the authors, informed by the FCC approach and the CFAM/CFIM frameworks. FCC, family-centered care; CFAM/CFIM, Calgary Family Assessment/Intervention Model; MV, mechanical ventilation.

The intensivist and RST aimed to reduce the patient's dependence on hands-on care; therefore, they initially pursued aggressive weaning from MV, which was also in line with the family’s wishes. They periodically reviewed the indications for further weaning attempts, but, up to DAI 70, no substantial improvement in the potential for liberation was noted. At that time, as progress and setbacks alternated, a structured, theory-guided approach based on FCC and the CFAM/CFIM was initiated, with HCU nurses leading the coordination of the interdisciplinary team. FCC provided a partnership ethos emphasizing flexible family presence, structured engagement, and standardized communication. CFAM guided the assessment across structure, development, and function, and CFIM aligned the interventions with the cognitive, affective, and behavioral domains with iterative reassessments. The dual-axis models of FCC and CFAM/CFIM are illustrated in Figure 1.

After introducing a nurse-led framework that combined both the FCC and CFAM/CFIM, planning, which had previously stalled, gradually began to show progress. Structured, family-inclusive conferences were held every 2-4 weeks, with ad hoc meetings at clinical inflection points. Each conference followed a standardized agenda with a teach-back loop to confirm shared understanding. The discussion items included respiratory and hemodynamic status, nutrition, sleep and psychological symptoms, rehabilitation progress, appropriateness of continuing weaning, and contingency planning. CFAM-informed assessment and CFIM-guided interventions were used to structure the work across the cognitive, affective, and behavioral domains, with explicit reassessment at subsequent encounters. Circular (reflexive) questioning at the bedside shifted focus from linear cause-effect explanations to relational and circular interaction patterns among family members, eliciting multiple perspectives and bringing core values to the surface. An option grid was used to structure a parallel comparison of home and facility discharge across two pathways-ongoing MV and cessation specifying requirements, anticipated burdens and benefits, and contingency plans. The grid detailed the required services, equipment, caregiver availability, expected burdens and benefits, and emergency response and procedures, and supported participation and collaboration during time-limited encounters. See Table 1 for the full item-level grid details.

**Table 1:** Option grid: home vs facility with MV Option grid comparing home-based care (with services) and facility-based care (with continued MV) for this case. Built for FCC-aligned, CFAM/CFIM-informed conferences using teach-back; maps options to the patient’s priority (“time to talk with family”) and outlines tasks, responsibilities, emergency pathways, visitation, rehabilitation, costs, and monitoring. Illustrative only—policies and coverage vary; decisions follow the agreed care plan. MV, mechanical ventilation; trach, tracheostomy; RST, respiratory support team; RH, rehabilitation; EN, enteral nutrition; ACP, advanced care planning; DNR/DNAR, do-not-resuscitate / do-not-attempt-resuscitation; ICU, intensive care unit; CO₂, carbon dioxide.

Domain	Home-based care (with services)	Facility-based care (continued MV)	Considerations/Notes
Caregiving skills & training	Tracheostomy site care/skin check; suction indications & asepsis; basic MV operations (alarms, emergency manual ventilation); secretion management (postural drainage, suction, humidification/hydration); enteral feeding prep/admin (gastrostomy care, clog prevention); oral care; emergency first steps; logging & reporting for home-visit nurses/physician.	Skilled nursing available 24/7; respiratory therapy/RST protocols for trach/MV; standardized suction/airway care; on-site emergency response; structured documentation.	Home requires caregiver competency and periodic retraining; facility centralizes expertise and reduces caregiver burden.
Equipment & supplies	Home MV; suction device; oxygen; humidification; pulse oximeter; backup power plan; consumables (circuits, filters, trach cannulas).	Institutional-grade equipment; supply chain handled by facility; redundant power and alarms.	Home needs reliable power and maintenance.
Staffing & emergency response	Family as primary caregiver; scheduled home-visit nursing; on-call support varies by facility; nighttime coverage contingent on services arranged. Home medical services (doctor's consultation) are also available, but only during the day. In addition, advanced medical treatment is not possible.	On-site clinicians; rapid response for acute changes; nighttime and emergency capacity. Even so, advanced medical treatment is impossible. When it comes to chronic care facilities, the medical treatment that can be done is even more limited.	Whether discharge is to a facility or home, responses to sudden deterioration are typically slower than in the hospital. Many long-term care facilities also stipulate, as a condition of admission, that they do not provide life-saving emergency interventions.
Costs & coverage	Device rental/consumables; home-visit nursing; rehabilitation; coverage via long-term care insurance/disability services; public subsidies and out-of-pocket ceilings.	Monthly fees; add-ons; bundled equipment/consumables; the cost varies greatly depending on the grade of the facility.	Compare copays/subsidies; perform a personalized cost projection before making a decision. In any case, it is assumed that they will be subject to physical disability certification and workers' compensation coverage.
Rehabilitation planning	Schedule tailored to sleep/feeding. As for rehabilitation, it depends on the amount of support from the family. There is also a home rehabilitation service, but it is limited to a very short time and the expected effect is small.	As a rule, facilities do not offer active rehabilitation. MV use generally precludes rehab-facility eligibility; The facility staff is barely able to maintain the MV and does not perform weaning in a desperate manner. In rare cases, daytime bed care is available.	Additional rehabilitation is generally limited and may not yield functional gains. The opinion of the Department of Orthopedic Surgery and Intensive Care at our hospital is that there is no prospect of further functional improvement.
Medical procedures & monitoring	MV weaning is premised on not doing it. No unsupervised weaning attempts (per family’s wishes). Tracheostomy care (cannula schedule, infection control); gastrostomy maintenance; monitoring for pleural effusion, aspiration; pressure-injury prevention; sleep/nutrition management. The following basically need to be done by family members, and you can regularly examine/consult with home visit nurses and doctors.	Under 24h supervision by nurses. Detection is faster when the condition worsens, but no further treatment is possible, so emergency transfer to an acute hospital is required. Therefore, there are many facilities that do not accept Code unless it comes out DNAR. In facilities with even lower medical throughput, the placement of devices such as MVs and catheters itself is often a reason for refusal of acceptance.	Whichever option is chosen, response to acute events may be delayed.
Discharge preparation & logistics	Home modifications; assistive device selection; backup power setup; caregiver roster and contingency plans. The patient (or family) is the contracting party with each service provider.	Admission coordination; handoff of nutrition and family visits plans; rehabilitation, recreation, and other care will be consulted with each facility.	Early planning reduces delays.
Values, goals & ACP	Document clarify ends versus means of care; update priorities; advance care planning; shared understanding of acceptable burden and risk.	As a rule, many facilities accept patients only with confirmed ACP (DNAR). The level of quality of life guarantee varies greatly depending on the facility.	At the very least, for the future of patients and their families, it is a priority to proceed with ACP confirmation.

Quantitative appraisal of weaning appropriateness was carried out by the RST, primarily based on mechanical power (MP) calculated from MV records around the time of tracheostomy. The patient’s MP was close to a reported cohort-specific threshold (approximately 256.5 J/min), placing the case in a prognostic gray zone; accordingly, we neither halted weaning immediately nor let it proceed without a time limit. In addition, spontaneous breathing trials were performed as part of the standard weaning assessment; however, full liberation from mechanical ventilation could not be achieved, and the weaning status remained unstable.

Nutrition and rehabilitation reframing addressed repeated feeding refusal, including enteral formulas. The intensivist, RST, dietitians, and nurses emphasized that adequate nutrition was vital to reduce pleural effusion and alleviate dyspnea regardless of whether weaning continued. Rehabilitation shifted from short-term functional gains to muscle relaxation, respiratory retraining, and psychological relaxation. The highest activity achieved during hospitalization was mobilization to an upright stance using a tilt table.

By DAI 80-90, bedside interviews clarified that the value underlying the wish for home was “to have time to talk freely with family.” In the subsequent family-inclusive conference at DAI 83, the intensivist and the RST explained that failure to achieve complete liberation would not necessarily impose immediate disadvantages. They also noted that reducing the stress of repeated trials could improve stability and safety for the patient. At that stage, no definitive indication for MV discontinuation was identified, and the team continued weaning on a conditional basis. However, the spouse reported that managing MV at home was not practical, and the team therefore explored facility options more thoroughly. Using the option grid increased transparency regarding required steps, caregiver burden, and emergency responses. The social workers confirmed the availability of facilities that accept patients on MV, the feasibility of flexible visitation policies that maintain family communication time, and eligibility for disability certification and workers’ compensation. If a facility offering flexible visitation was available and other requirements could be met, the family expressed a preference for transfer with ongoing MV. This differed from the prior plan, which had identified home discharge after successful weaning as the ideal goal. A few days later, the family conveyed the conference’s provisional decisions to the patient. The patient stated that continuing to push himself with further weaning and RH was burdensome, that he felt safer with the MV, and that he would accept transfer to a facility if he could regularly meet with his family while on MV. A medical social worker then identified and secured a facility that met the specified conditions, and disability certification and workers’ compensation were approved and implemented. The plan regarding weaning cessation and subsequent discharge coordination was finalized. MV settings were titrated for comfort and safety consistent with the receiving facility’s criteria. Eventually, the patient was transferred from an acute hospital under MV at DAI 166. After his discharge, the family later visited the acute care hospital to thank the staff who had cared for him. His wife, in tears, said, “At first, our children wanted to honor my husband’s wishes and hoped he could be cared for at home, and I felt the same. But I knew I could not care for him by myself and was torn between not betraying everyone’s expectations and the dilemma of placing him in a facility.” Like this, the patient and his family expressed relief that a plan consistent with his values and safety could be implemented.

Discharge to a nursing home (a long-term care facility where nurses are continuously present and family members can visit freely) was decided, and, to reorganize his care, a short-term bridging admission to a chronic care hospital was arranged in advance. For the discharge summary, the medical aspects were prepared by the attending physician and the ICU physician, whereas the care-related aspects were prepared primarily by the nurses and the RST.

The care to be handed over consisted of maintaining essentially fixed MV settings (assist/control-pressure control mode, inspiratory pressure 10 cmH₂O, positive end-expiratory pressure 5 cmH₂O); endotracheal suctioning at least once every eight hours and additionally whenever the patient expressed discomfort; repositioning every two hours; elevating the head of the bed as much as possible during the daytime; and providing oral care. For episodes of dyspnea, the planned responses included attentive listening to the patient’s complaints and, as appropriate, (1) a temporary 100% oxygen flush followed by endotracheal suctioning, (2) gentle reassuring touch, (3) adjustment of the pillow position, which had previously been effective in relieving his dyspnea, and (4) consulting the hospital affiliated with the facility if abnormal vital signs or repeated MV alarms occurred.

Table 2 shows the chronology of clinical events, nursing and team interventions, and milestones in consensus building.

**Table 2:** Event–intervention–decision timeline DAI, days after injury; MV, mechanical ventilation; CO2, carbon dioxide; HFNC, high-flow nasal canula; ICU, intensive care unit; HCU, high-care unit; NPO, nil per os. RH, rehabilitation; ROM, range of motion; QOL, quality of life; PT/OT, physical/occupational therapy; FCC, family-centered care; CFAM/CFIM, Calgary Family Assessment/Intervention Model; EN, enteral nutrition; IC, informed consent

Event category	DAI 1–30	DAI 31–60	DAI 61–90	Day 91– Discharge
Respiratory	DAI 1: ICU admission; MV initiated. DAI 4: early tracheostomy. DAI 5: Weaning began. DAI 26: Temporary liberation success. Tried the speech valve but couldn't stand it.	DAI 48: Due to hypoalbuminemia, “CO_2_ narcosis” due to pleural effusion, continuous withdrawal time was only a short time (5 days). During the time of MV liberation, T-piece or HFNC management. Basically, it was general ward management, but when the condition deteriorated, ICU or HCU readmission. DAI 90: Weaning abandoned.		Weaning was discontinued and the settings were optimized for facility transfer. Handoff to the receiving facility.
Nutrition	DAI 1-3: NPO. DAI 4: EN initiated (900 Kcal). DAI 9: Oral intake also started. Shifted to oral intake only, but Unable to secure sufficient nutrients, composited EN and oral intake (EN: 1000-1600 Kcal, Oral: 100-300 kcal).	Kcal/protein up‑titration. DAI 33: EN denial. DAI 48: 1440 Kcal	EN and oral intake rejection often persists. DAI 67: Reach full dose for EN requirements (2210 Kcal).	Since the reason for refusal of EN and oral intake was abdominal distension, the main time of administration of EN was changed to nighttime. DAI 123: Gastrostomy DAI 128: EN 2210 Kcal invariant. Oral intake: only when the person wants it (family members give the food they want)
Albumin	DAI 1 to 8: decreased 4.4 to 2.1 g/dL. DAI 8 to 31: 1.9 to 2.2.	DAI 31 to 60: increased 1.9 to 2.7 g/dL.	DAI 61 to 90: increased 2.0 to 2.6 g/dL.	DAI 91 to 120: increased 2.3 to 2.9 g/dL. DAI 150: 2.9 g/dL.
Mental health	DAI 9: Psychological instability began to manifest itself (insomnia, anxiety). DAI 15: A multidisciplinary mental health liaison team was consulted. DAI 20: Became depressed and was prescribed antidepressants.	DAI 33: Insomnia persists; increasing prescription of antidepressants. DAI 48: Increased sleeping pills and psychotropic medication were prescribed because symptoms did not improve and insomnia symptoms were particularly strong.		DAI 97: Adding a new type of psychotropic medication. DAI 111: Increased the dose of sleeping pills for persistent depression and insomnia. DAI 125: No change, schizophrenia drugs added. DAI 138: No change, schizophrenia drugs and sleeping pills added. DAI 145: Improving Insomnia. DAI 150: Depressive symptoms also tend to improve.
Rehabilitation	DAI 2: PT intervention; ROM training with limited range of activity on the bed. DAI 6: OT intervention; Bedup limited to 30 °. DAI 7: OT intervention; Bedup limited to 60 °. DAI 10: Permission to leave the bed with a neck protection fixture. DAI 15: began to refuse for RH; took a walk around the hospital while lying on the bed.	DAI 49: Went to ICU due to deteriorating condition. DAI 53: To HCU. Leave the bed according to the patient's mood. RH reopened; Start from the bed.	DAI 61: Tilt table was used to try forced standing. DAI 62: Dopaminergic medications were prescribed because orthostatic hypotension and vagus nerve reflex occurred.	DAI 68: Transferred to a wheelchair. Cyclic dynamic fluctuations have gradually become an improving trend. DAI 85: No longer need to wear a fixator for neck spine protection. Stable participation in RH (EN refusal is also almost gone). The rehabilitation plan was taken over.
Discharge decision support	A state in which both patients and their families do not understand the situation. DAI 10: The patient's mental state became unstable, and the family was also upset by this. IC was performed when needed to communicate the unplanned/structured patient situation.	Regarding discharge from the hospital and future life, medical staff talked about it as a topic, but they couldn’t afford it to think about it. The patient expressed his desire to go home. Family also wanted to follow this wish.	The important value of patients is "guaranteed time to talk to their families without hesitation" became clear between patients, nurses, and families. DAI 83: FCC and CFAM; the first planned conference based on CFIM. DAI 90: Family decides to cancel MV weaning.	Regular meetings of family participation conducted using a structured and standardized agenda and teach-back. It was found that social resources in terms of money and nursing care support were available for almost full support. Therefore, could decide whether discharge to facility or home. Unified plan communicated to receiving facility. After that, the family informed the patient about the policy of discontinuing weaning and transferring the facility step by step, taking into account the patient's mental state. The patient also agreed to this proposal because he did not want any more Weaning and did not want to cause trouble to his family in terms of caregiving because he was free to visit his family even if he was not at home. DAI 166: Transfer from acute hospital. Discharge to a nursing home (a long-term care facility where nurses are continuously present and family members can visit freely) was decided, and, to reorganize his care, a short-term bridging admission to a chronic care hospital was arranged in advance.

## Discussion

This case shows that a nurse-coordinated, equal-partnership approach grounded in FCC and the CFAM/CFIM model reframed a binary, physiology-first target into a values-first plan while preserving clinical prudence. After the ICU phase, the approach began mainly in the HCU and was carried forward to the general ward, where nurses continued to apply FCC and the CFAM/CFIM model to guide bedside practice and engagement with the family [1,2,8,9]. CFAM structures assessment across structure, development, and function, and CFIM aligns interventions with cognitive, affective, and behavioral domains with reassessment to foster new interaction patterns [3,4].

These methods shifted conversations away from linear cause and effect toward relational interactional patterns within the family, consistent with the reflexive questioning approach described by Tomm, which is designed to elicit new meanings and promote change [5]. The medical team held brief, structured family interviews at regular intervals and used teach-back techniques to ensure shared understanding. This follows guidance on brief, structured family encounters and standardized communication in critical care and helps build shared understanding and support decisions [1,8]. Through this process, we clarified that “home” symbolized the patient’s core value of having time to talk freely with family. That value then became the anchor for subsequent decisions.

Option grids were used as encounter-time tools to make options visible. They organize side-by-side comparisons around patients’ frequently asked questions and support shared decision making in routine encounters [7,10,11]. In this case, the option grid presented home and facility discharge options side by side for both scenarios-continued MV and discontinuation. Clarifying requirements, burdens, and benefits, and emergency responses allowed movement away from a wean-or-fail frame toward a plan that matched the articulated value while maintaining safety. It is important not only to conduct structured discussions and present option grids, but also to use circular questioning to continuously assess how the patient and family are perceiving and receiving the situation. In this case, there was no direct involvement of an ethics team; however, if these processes are neglected, later ethical dilemmas may arise even when a common goal regarding the discharge destination has been formally agreed upon, particularly when the actual environment of the receiving facility diverges from what was initially envisioned. Therefore, from an ethical standpoint as well, it is necessary to evaluate the validity of the decisions reached throughout the process.

In tracheostomized prolonged weaning cohorts, a threshold near 256.5 joules per minute has been reported, which places values near that level in a prognostic gray zone [12]. However, MP correlates with ventilator outcomes, but absolute thresholds vary by population and the calculation method employed, and should be interpreted cautiously [13]. The threshold had been reported in a study conducted in the same institution, increasing its relevance to local practice. Therefore, because the patient’s value lay within the gray zone around that threshold, interpretation was complex and favored the use of parallel planning. As part of the standard weaning assessment, SBT recommended by Boles et al. was also performed; nonetheless, complete liberation from mechanical ventilation could not be achieved, and the weaning status remained unstable [14]. This approach prevented premature discontinuation and unchecked trials while remaining consistent with nonmaleficence and respect for values.

Translating the value into a feasible plan required meeting several enabling conditions. Nurses coordinated the interdisciplinary team with the family to secure a facility that could continue care under mechanical ventilation, confirm flexible visitation to preserve family communication time, and obtain coverage through disability certification and workers’ compensation [1,2,8,9]. This coordination supported an equal partnership between the medical team, including nurses, and the family, translating the patient’s articulated value into a concrete, feasible plan. The final decision to transfer to a facility with flexible visitation while continuing MV was jointly chosen by the patient and family as the preferred goal, representing a measured course rather than a cessation of effort. These points are crucial for the clinical application of the two-axis nursing theory model and are also essential when considering its generalizability. What made this possible, in concrete terms, was a combination of cyclic communication with the family, structured regular interdisciplinary discussions, and the use of an option grid to organize and compare possible trajectories. Through these processes, the patient’s core values were repeatedly elicited, shared, and clarified, and the multidisciplinary team was encouraged to consider, from the perspective of each profession, what could be done to move toward the realization of those values. At such times, it is crucial that each professional does not allow a provisional goal (for example, discharge to a facility with ongoing MV) to bias or truncate their own specialist assessment. For instance, the RST should maintain a commitment to thoroughly and multidimensionally evaluating the potential for MV liberation until the end of the process. The information and insights obtained in this way are then shared through conferences that explicitly include the family, and repeating this cycle appears to be highly effective. Although the magnitude of benefit will naturally be influenced by individual case characteristics and local context, this approach appears applicable to the provision of seamless care across transitions from critical care areas to general wards in other institutions. Standard nursing care is not inherently inadequate; however, when care is delivered in a non-planned, non-structured, and non-dialogical manner, it becomes difficult both to reach an optimal goal and to evaluate what constitutes “the best” outcome. Even if an optimal goal is achieved under such conditions, it tends to be the product of chance, without deliberate reflection and with limited reproducibility. What is essential is that the healthcare team and the patient and family intentionally and collaboratively reflect on what the best attainable goal should be and purposefully work toward it. From a theoretical standpoint, it is noteworthy that this two-axis nursing theory approach can be implemented in virtually any disease condition, patient context, or healthcare setting.

There are several limitations to this case report. This is the report of a single case from one institution; therefore, the generalizability of the information herein is limited. Processes and resources that enabled implementation, including the availability of a facility accepting MV, flexible visitation policies, and coverage through disability certification and workers’ compensation, may not be uniformly accessible in other settings. There is no comparator group, and the qualitative effects of structured communication, circular questioning, and option grids may be subject to selection and documentation bias. Moreover, in this case, no long-term follow-up was conducted after discharge from the acute care hospital. Although this intervention appeared to have a favorable impact on the seamless delivery of nursing care and team-based medical practice across wards within a single facility, it remains unclear whether its beneficial effects would continue to extend to inter-hospital collaboration beyond the acute care setting and to long-term care environments. Nonetheless, the process architecture appears to be reproducible to some extent. This dual-axis model of FCC and the CFAM/CFIM model may provide practical heuristics for similar prolonged weaning cases.

## Conclusions

A nurse-coordinated FCC and CFAM/CFIM approach can transform stalled decision-making during prolonged MV into value-congruent, sustainable care plans by anchoring decisions in what matters to patients and families while preserving clinical prudence. Future work should prospectively evaluate this process using quantitative, long-term outcomes. Developing brief, context-specific option grids and practical training for bedside staff may support wider adoption.
